# High-energy synchrotron X-ray tomography coupled with digital image correlation highlights likely failure points inside ITER toroidal field conductors

**DOI:** 10.1038/s41598-021-01999-5

**Published:** 2021-11-30

**Authors:** Ryan Warr, Matthew C. Jewell, Neil Mitchell, Alexander Rack, Jack Swanson, Vladimir Tronza, Robert Cernik

**Affiliations:** 1 Department of Materials, University of Manchester, Oxford Rd, Manchester, M13 9PL UK; 2 The Sir Henry Royce Institute for Advanced Materials Research and Innovation, Manchester, UK; 3 Materials Science & Biomedical Engineering, University of Wisconsin-Eau Claire, Eau Claire, WI 54702 USA; 4 ITER International Organization, Route de Vinon-sur-Verdon, 13115 Saint-Paul-lez-Durance, France; 5 ESRF –The European Synchrotron, 71, Avenue des Martyrs, 38000 Grenoble, France

**Keywords:** Energy science and technology, Engineering, Materials science

## Abstract

Two sections of heat-treated (HT) and non-heat-treated (NHT) Cable-in-Conduit Conductor (CICC) of a design similar to the ITER tokomak have been imaged using very high energy X-ray tomography at the ESRF beamline ID19. The sample images were collected at four temperatures down to 77 K. These results showed a greater degree of movement, bundle distortion and touching strands in the NHT sample. The HT sample showed non-linear movements with temperature especially close to 77 K; increasing non-circularity of the superconducting fibre bundles towards the periphery of the CICC, and touching bundles throughout the CICC. The images have highlighted where future design might improve potential weakness, in particular at the outer perimeters of the conductor and the individual sub-cable, ‘petal’ wraps.

## Introduction

The ITER tokamak project, first proposed in 1985, aims to provide both scientific and technological insight into the potential of fusion power as a means of global power generation^1^. Superconducting coils are used to generate the magnetic fields required to sustain the 150,000,000 °C plasma necessary for fusion to occur. ITER uses superconductors based on Nb_3_Sn intermetallics for its Toroidal Field (TF) Coils and Central Solenoid (CS). These components of the ITER magnet system will be exposed to high magnetic fields that will reach up to 13 T^2^. Nb_3_Sn is the only superconducting material that is both able to carry the required current in such magnetic fields and also has well industrialised manufacture. The latter makes a procurement of large amounts of material, a few hundred tons in the case of ITER, feasible.

A negative aspect of the Nb_3_Sn intermetallic nature is its extreme brittleness under tensile strain that leads to fractures in Nb_3_Sn filaments and, as a consequence, decreases the superconducting performance^3^.

The TF and CS magnets are made of a Cable-in-Conduit Conductor (CICC) which is a multistage cable of copper and Nb_3_Sn strands twisted together and enclosed into a stainless steel tubular jacket. The full 5-stage process of CICC manufacture has been described in detail elsewhere^4,5^. Over 1000 strands, slightly above 0.8 mm in diameter, form the TF cable. In its turn each strand contains thousands of microscopic (a few µm in diameter) superconducting Nb_3_Sn filaments. In order to provide necessary cooling of the CICC, a cooling channel made of an open steel spiral is placed in the centre of the cable. The cable itself is porous with a void fraction of about 30%, which allows coolant to reach every single superconducting strand. As part of the production process for CICCs, the wound bundles are heat treated in order to form the superconducting properties of Nb_3_Sn. The, as delivered, Nb_3_Sn strands have a twisted arrangement around their own axis, with a pitch of approximately 15 mm. The Nb_3_Sn strands used in the production of the TF conductors are fabricated using both the ‘bronze-route’ and the ‘internal tin’ processes^6,7^. The superconducting Nb_3_Sn composition only forms in the individual strands after the heat treatment of the whole assembled CICC.

For a long time it has been known that Nb_3_Sn conductors demonstrate degradation of their performances due to micro cracking that occurs in brittle superconducting filaments^8^. This was usually observed when strands deflected under cyclic electromagnetic (EM) loads that simulated ITER operational conditions. Multiple tests were carried out on ITER conductors and showed that the performance of the conductors exposed to EM cycles tend towards stabilisation and the progressive degradation becomes less significant^9^. This positive conclusion though had at least one flaw. If a conductor had undergone a thermal cycle, known as the warm-up and cool-down (WUCD) process, i.e. warm up to room temperature with following cool down to 4 K, the degradation was observed to restart under EM cycling^10^. Stabilization of conductor performances vs WUCD cycling, or rather a combination of WUCD and EM loads, is achievable but consumes a significant portion of the initial performances as the degradation is irreversible.

A practical solution was found for the CS CICC which had also previously demonstrated significant degradation with cycling. A smaller cable twist pitch was used which increased the cable density^11^. This new and denser cable demonstrated no degradation as the strands had better mechanical support with less possibility to move and deflect^12^.

Despite this practical solution, there are long-term use concerns for the durability of CICCs in operation where they will be subjected to thermal/EM cycling and intense neutron radiation. It is therefore still important to get a clear understanding of a mechanism of degradation caused by WUCD. Previous investigations have been conducted to measure the stress and strain induced on the superconducting strands as a result of both thermal and electromagnetic loading, implementing techniques such as metallography^13^ and neutron diffraction^14,15^. However, no attempts have been made to date to visualize strand movements during WUCD, and to see directly what is happening inside the ~ 5 cm diameter stainless steel circular jacket of a fully fabricated TF conductor. In this experiment we have used high energy, high resolution X-ray tomography to observe behaviour of cable and individual strands, produced using only the ‘internal tin’ process, in a cross-section during WUCD. The results of the investigation have led to a better understanding of the mechanism that causes degradation of the superconducting performance in CICCs and will inform future designs looking ahead to full scale power production.

The high energy X-ray beams available at the ESRF were used in order to conduct high spatial resolution imaging of sections from the ITER TF conductors. By conducting micron-scale imaging, a full cross-section of the conductor was observed, providing contrast between individual strands in a non-destructive manner. The samples were imaged over a range of temperatures down to 77 K, where the cables experience approximately 85% of the contraction expected during routine thermal cycling in ITER^12^. We therefore believe that the imaging experiment described here gives a realistic picture of the strand movements inside a metre-long section of a heat-treated and non-heat-treated section of TF CICC. We observed the degree of thermally induced strand motion allowing potentially weak points within the conducting coils to be highlighted.

## Experimental

**Figure 1:**
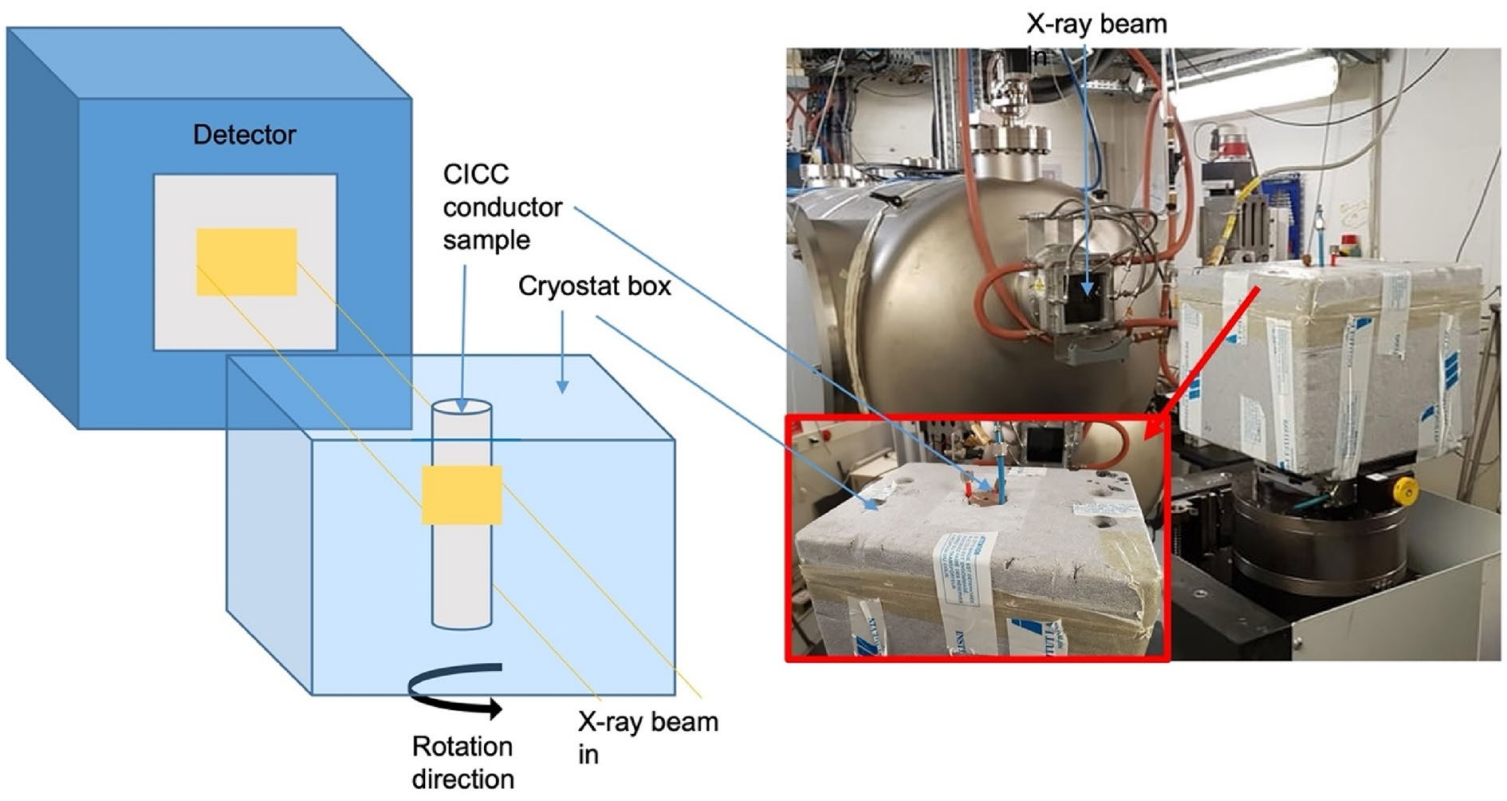
Synchrotron beamline configuration. (Left) Schematic of the scanning procedure for the CICC sample, placed within a polystyrene container and secured in place on the rotation stage. A rectangular X-ray beam slightly wider than the sample passed through a vertical section of the sample to be recorded by the detector (not to scale). The sample was rotated (black arrow) by the rotation stage as multiple projection angles were recorded. (Right) Photo of the experimental set-up. A close-up of the top of the polystyrene container with fixed CICC sample is included (red box insert).

Two samples, 4.4 cm in diameter and of different lengths were cut from the as manufactured length of CICC. One sample was heat treated (HT—44 cm length) to react the superconducting strands; the other sample was not heat treated (NHT—20 cm length). The standard heat treatment approach involves tens of hours of initial treatment between 100 and 400 °C, before a cycle of approximately 200–250 h at 650 °C^4^. The two samples were scanned to form images on beamline ID19 of the European Synchrotron Radiation Facility (ESRF, France). ID19 is uniquely well suited for studying samples of these dimensions and densities: having a large sample-source distance (~ 150 m); a maximum horizontal beam size of approximately 5 cm, and a vertical useable beam size of 2 cm. Data were collected in white/pink beam mode: the radiation emitted from a W150-type wiggler (at minimal gap of 26.5 mm) was attenuated by absorbers (5.6 mm Al, 2.3 mm W, 14.2 mm Cu, 0.7 mm Mo, 0.28 Au, 1 mm C) to significantly harden the beam. The resulting X-ray spectrum at the detector had a (broad) peak around 240 keV, the photon flux density was sufficient to record tomographic scans in less than 0.5 h. In order to further increase the contrast, a propagation distance between sample and detector of 13 m was chosen: refraction of X-rays at interfaces inside the specimen boost their visibility, so-called hard X-ray phase contrast. The detection system was made by ESRF with a 2000 µm-thick LuAG:Ce scintillator lens-coupled (demagnified) to a sCMOS-based camera. In each case, the sample was exposed to an X-ray beam of size 5 cm by 1 cm, allowing the full cross-section of the conductor sample to be scanned for a 1 cm thick section in a single acquisition. A schematic of the set-up for the tomographic imaging is shown in Fig. 1.

In order to observe the movement of the strands inside the CICC steel jacket as a function of temperature the conductor was placed in a polystyrene cryostat box containing liquid nitrogen. The entire cryostat was filled with liquid nitrogen such that the imaged section of the conductor was fully submerged. In each case, the 5 cm by 1 cm X-ray beam passed through the approximate centre of the cryostat box, corresponding to the centre of the NHT conductor and at around 75% depth of the HT conductor. The sample was cooled to 77 K where the selected 1 cm section was scanned. The liquid nitrogen was then boiled off at a controlled rate to increase the sample temperature. Two more sections were scanned at 161/173 K and 223/225 K prior to the final room temperature scan. During imaging, the conductor was fixed in the cryostat to prevent movement during the angular movement for each projection. The conductor was fixed in position using tape and low-temperature wax, combined with its own weight. Additional wires ensured the tightness of the cryostat during sample rotation, such that no motion artefacts were observed during image acquisition (see Fig. 1). In total 4 scans at different sample temperatures were acquired for each sample, ranging from 77 K up to room temperature. 5000 projections were recorded over a sample rotation of 360°, with a total scanning time of 45 min per scan. Both the cryostat box and conductor sample rotated together during scanning. The temperature range during each scan was not more than 10 °C as measured by the thermocouple placed directly on the sample. We have assumed that the relatively small thermally induced fibre movements during a scan will be negligible.

### Image reconstruction

**Figure 2:**
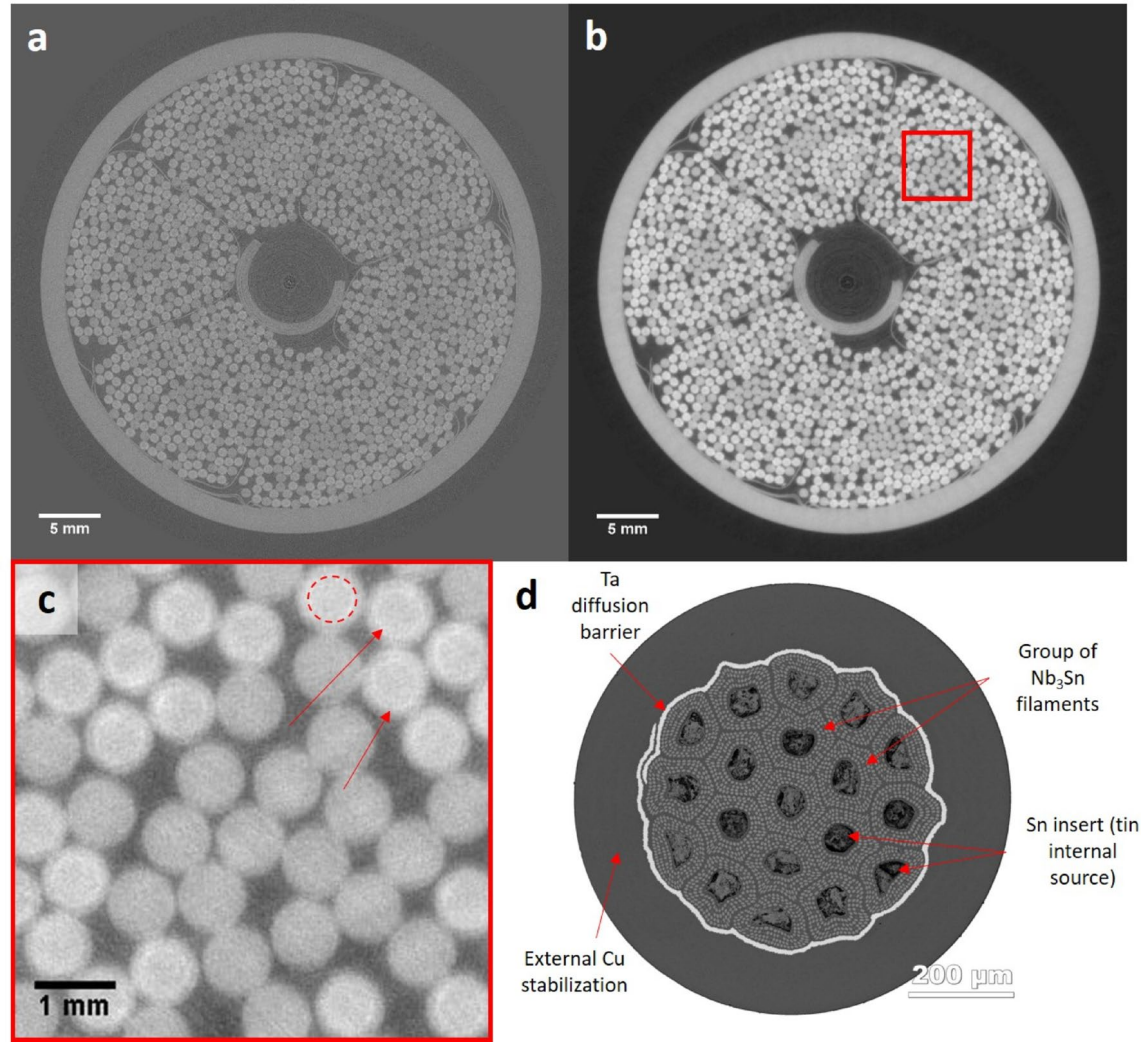
Effect of filtration on reconstructed conductor. (**a**) An original reconstructed image slice of the heat-treated conductor prior to filtration. (**b**) The identical image slice upon application of the phase retrieval filter based on Paganin’s approach. (**c**) An expanded view of the conductor bundles (red box) in (**b**). Pure copper strands appear darker in colour than Nb_3_Sn strands. In addition, the resolution allows the Ta diffusion barrier to be seen as a circular perimeter (red-dashed circle) around the Nb and Sn components (red arrows for other examples). (**d**) Diagram detailing the structural configuration of a single heat-treated Nb_3_Sn strand, where some Sn has diffused into the Nb filaments to begin formation of the Nb_3_Sn composite.

Tomographic reconstruction of the radiographs was performed using filtered back-projection, producing 400 image slices across a 1 cm thick section of the samples, with a voxel size of 21.8 µm when accounting for geometric and optical magnification of the sample. Spatial resolution is estimated to be approximately 60 µm. During post-processing, a Paganin filter^16^ was applied to both sets of reconstructed volumes. Prominent ring artefacts were observed in the image slices, potentially due to non-linear pixel response. These were in part reduced on application of the filter along with improved contrast, as shown in Fig. 2.

Figures 2a and b illustrate that the solid copper and Nb_3_Sn strands were clearly differentiated after the application of a post-processing filter. The high-resolution synchrotron images shown in Fig. 2b and c reveal the internal structure of the assembled conductor. The fibre bundles are a mixture of pure copper (darker) and Nb_3_Sn (lighter) strands. Given the high spatial resolution of the images, it is possible to observe the tantalum (Ta) diffusion barrier as a circular perimeter within individual Nb_3_Sn strands (Fig. 2c). The Ta barrier lies between the external Cu stabilisation layer, and the internal composite superconducting core, consisting of thousands of Nb filaments and an internal tin matrix, a common design for creation of Nb_3_Sn strands using the ‘internal tin’ process. Figure 2d shows a diagram of a single Nb_3_Sn strand after application of heat treatment, where Sn has started to diffuse into the Nb to form the Nb_3_Sn composite. As expected, the Ta barrier can be seen in both the heat-treated and non-heat-treated samples.

The reconstructed image slice also shows part of the central steel support spiral; the thin metallic foils holding the bundles in six ‘petals’; the outer steel jacket and the space between components for the cryogenic fluid to flow.

### Analysis

**Figure 3:**
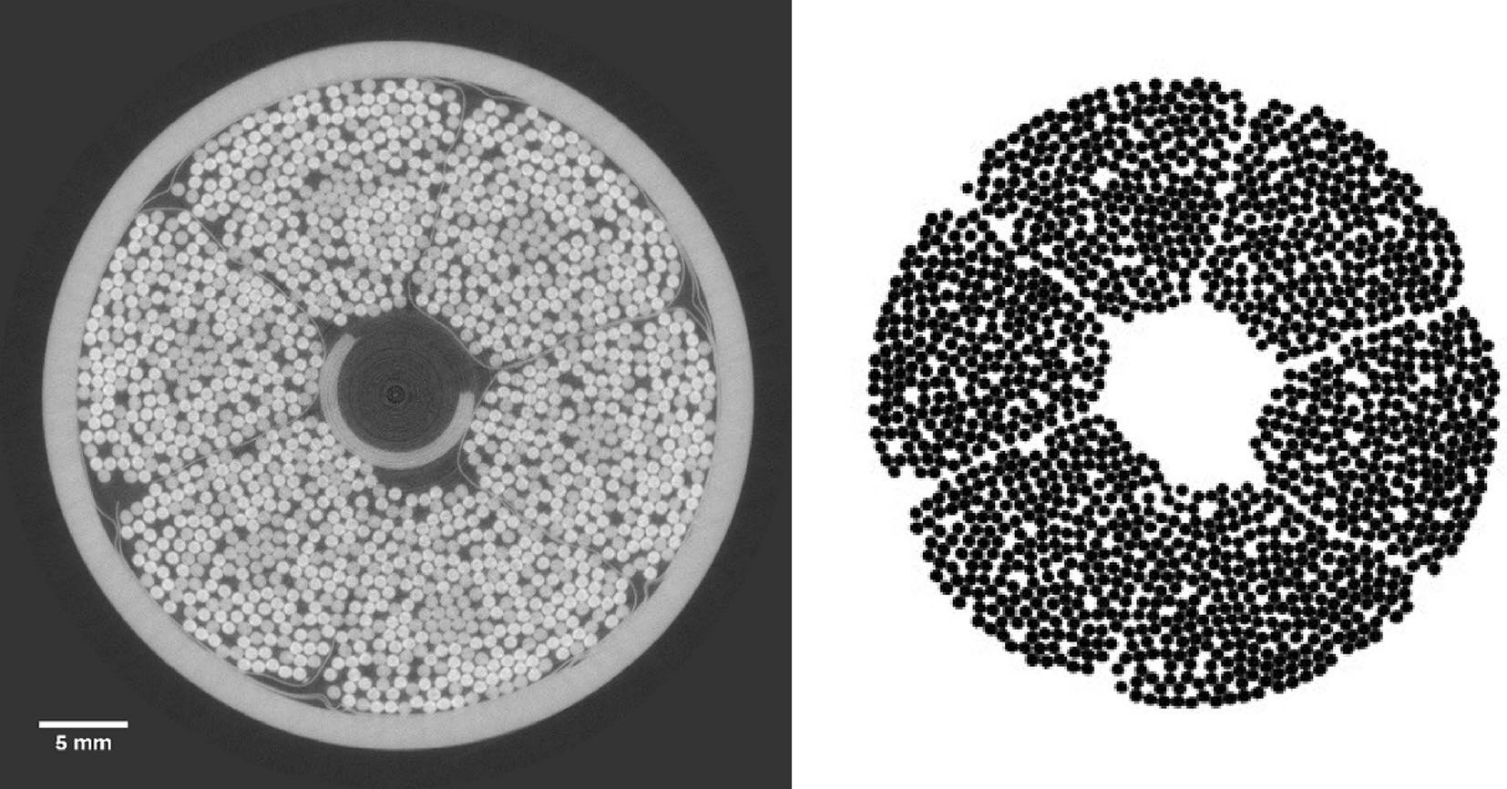
Thresholding and segmentation procedure of conductor. (Left) A reconstructed image slice of the conductor, prior to thresholding. (Right) A binarised image of the superconducting bundles, isolated from all other components.

Two reconstructed image slices were taken from both the heat-treated (sections A1 and A2) and non-heat-treated conductors (sections B1 and B2) for further analysis. These corresponded to vertical depths of approximately 2 mm and 5 mm into the 1 cm thick reconstructed image volume. In order to observe individual fibre bundle movements, a thresholding regime was implemented, such that all other components of the conductor (including the steel cooling spiral, outer jacket and petal envelopes) were removed. Figure 3 illustrates an example of the thresholding technique, producing a binarised image from a reconstructed slice. Images corresponding to the same slice at each temperature were checked to correct for possible sample misalignments between sequential scans. A full set of images for each slice of the sample is shown in the supplementary material together with four videos of the temperature induced fibre movement for samples A1, A2, B1 and B2.

In order to evaluate the degree of strand movement as a function of temperature, digital image correlation (DIC) was implemented to quantify the spatial variation of individual strands. DIC has become a standard tool in measuring local strain and displacement across successive 2D image slices, with optical and electron microscopy examples of fields where the technique is used extensively^17^.

## Results and discussion

By recording the position of each individual fibre as a function of temperature, the displacement was quantified by comparing values from one reconstructed slice to the next, from liquid nitrogen temperature up to room temperature. The results allowed observation of local regions in the samples where fibre bundle displacement was significant. Further patterns were then identified based on the effects of both heat treatment and temperature. Calculated averages across the three incremental temperature steps highlighted these effects, with full results and associated errors from the DIC method shown in Table 1.

**Table 1:** Average strand movement as a function of temperature. (Top) The measured values determined through DIC for the heat-treated sections A1 and A2, with associated standard deviations. (Bottom) The corresponding values for the temperature ranges measured at each step of the non-heat-treated sections B1 and B2. The steps are comparisons of the relative movement of the conductor strands within the temperature intervals shown. The final line is the overall average movement between steps.

Temperature step/K	Heat-treated section A1		Heat-treated section A2	
	Avg. movement/mm	Standard deviation/mm	Avg. movement/mm	Standard deviation/mm
1 77 → 161	0.093	0.046	0.093	0.047
2 161 → 225	0.063	0.016	0.022	0.011
3 225 → 300	0.060	0.029	0.043	0.019
Ave. 1 → 2 → 3 → 4	0.072	0.030	0.053	0.026

Temperature step/K	Non-heat-treated section B1		Non-heat-treated section B2	
	Avg. movement/mm	Standard deviation/mm	Avg. movement/mm	Standard deviation/mm
1 77 → 173	0.037	0.022	0.079	0.031
2 173 → 223	0.071	0.033	0.118	0.050
3 223 → 300	0.127	0.076	0.193	0.091
Ave. 1 → 2 → 3 → 4	0.078	0.044	0.130	0.057

**Figure 4:**
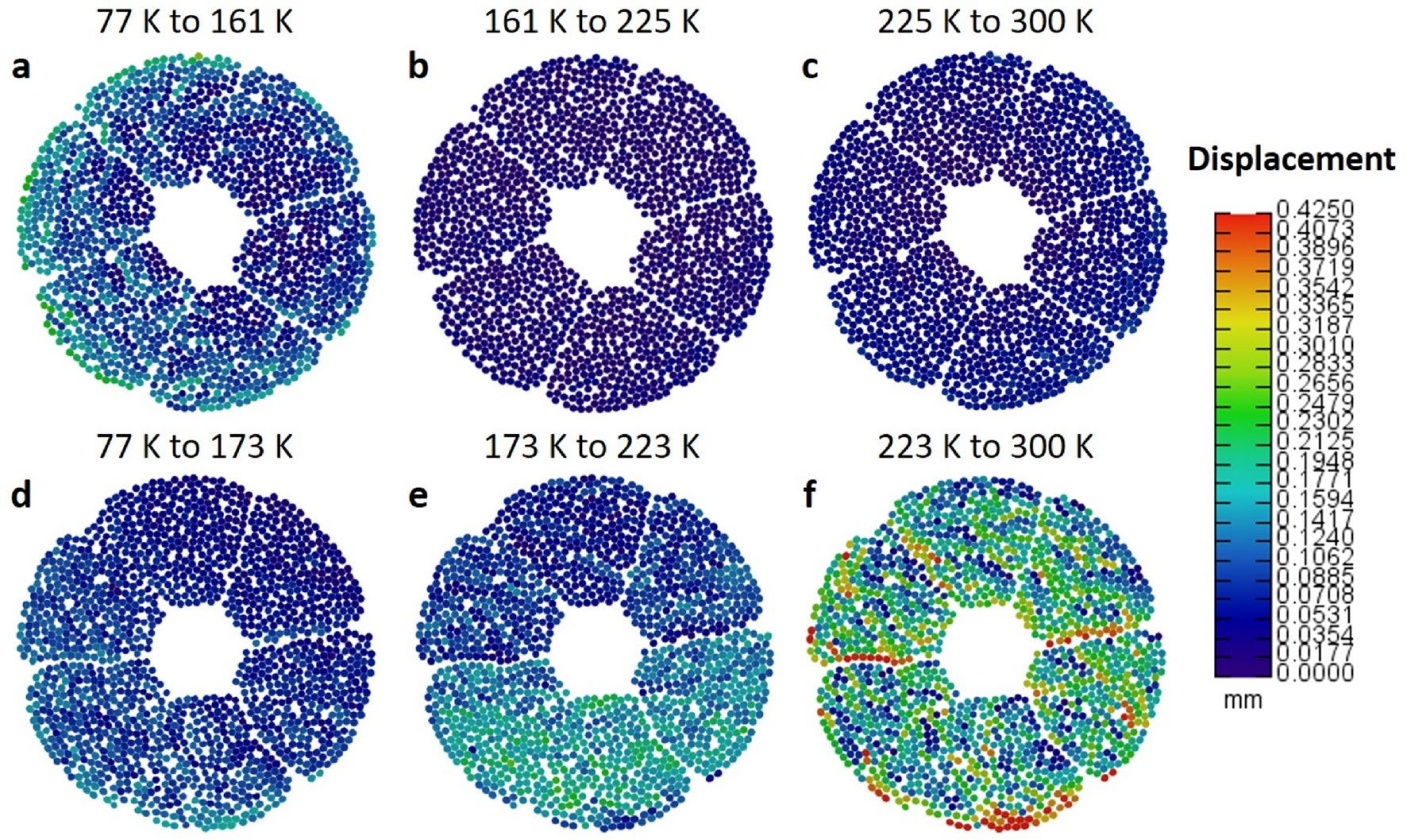
Fibre bundle movement as a function of temperature. (**a**–**c**) Relative movement of strands for section A2 of the heat-treated (HT) conductor. A colour scale is used to map and quantify the degree of movement between each step in temperature, with differences measured in mm. (**d**–**f**) Equivalent colour maps for the section B2 of a non-heat-treated (NHT) conductor.

The results for Sections A2 and B2 are shown in Fig. 4. Direct comparison of the colour maps indicated a significant difference in the degree of strand movement, particularly as the samples warmed to room temperature at the end of their thermal cycle. The employment of heat treatment provided a large reduction in the movement of each fibre bundle at these higher temperatures, as shown by comparison of Fig. 4c and f. An average movement of 0.043 mm was measured across the final temperature step for the heat-treated sample, significantly lower than the average displacement of 0.193 mm calculated for the non-heat-treated equivalent. Slightly increased movement of the fibre bundles were measured in the first temperature step of the heat-treated sample compared to its non-heat-treated counterpart (Fig. 4a,c), as each conductor warmed from liquid nitrogen level. This was also observed in Section A1 and B1, as indicated by Table 1.

The use of displacement colour maps provides a unique way to examine the regions of the CICC most at risk of higher distortion across the temperature range. For both the HT and NHT conductors, areas of higher displacement occurred close to the interface with the stainless-steel jacket, with the differences in thermal expansion coefficients between the conductor fibres and supporting jacket influencing the degree of movement. Significantly, in the absence of heat treatment (Fig. 4d–f), ‘lines’ of greater movement were observed at the interior ‘petal’ edges, where bundles were in contact with the sub-cable wrap. Figure 4f strongly illustrates such regions of weakness, compared to its relatively stable counterpart in Fig. 4c.

The fibre bundle movement was also analysed by examining the displacement per unit temperature. This was thought to be important given the non-uniform experimental temperature steps. We observed opposing trends between the HT and NHT conductors. For both sections of the NHT conductor we observed a sharp rise in displacement following the temperature step from 77 to 173 K, with only a small further increase in movement during the final transition to room temperature. In contrast, only the final temperature step of the HT conductor from 223 to 300 K (section A2) showed a difference in the general, near linear, trend observed for the heat-treated conductor, where average movement (per unit K) fell as temperature rose. The results per unit K also show a consistently higher degree of movement at lower temperatures for the HT and NHT samples despite the addition of heat treatment to one sample. The effectiveness of the heat treatment procedure may therefore be questioned in the low temperature regime of the thermal cycle.

**Figure 5:**
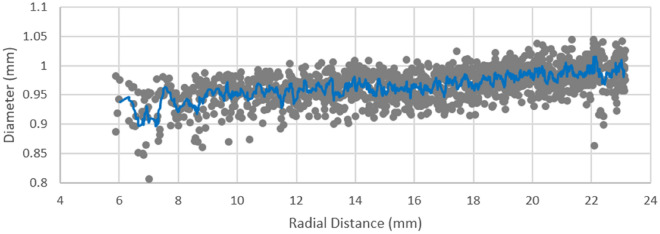
Fibre bundle effective diameter as a function of radial distance from the centre of the CICC. A gradual rise in bundle diameter is observed for the heat-treated sample with increasing distance from the jacket centre of mass.

A further proxy for potential degradation of superconducting wires within the CICCs is strand bending when exposed to test conditions. While previous experiments have quantified the strain on individual strands under electromagnetic loading conditions^18^, here we illustrate the effect of the integrated cable twist pitches on the angle that the fibres make with the transverse cross-section of the conductor. A measure of this geometric effect was evaluated through the degree of variation in the effective fibre bundle diameter in the tomographic transverse cross-section. Larger values of effective diameter (as measured by calculating the area of each fibre and applying a circular approximation to the shape) are evidence that the strand is at a steeper angle with respect to the conductor axis. The effective diameter of each individual fibre bundle was measured as a function of its radial distance from the external steel jacket centre of mass. The measurements for each conductor were assessed and plotted for data acquired at liquid nitrogen temperature. As illustrated in Fig. 5, an increase in effective fibre bundle diameter was observed for bundles closer to the jacket interface. A lower-bound average of 0.90 mm diameter at approximately 7.2 mm radial distance, compared to a high of 1.01 mm diameter at 22 mm radially. This indicates that the fibres near the jacket interface have a different local geometry than those near the conductor centre and can be expected to deform differently under electromagnetic forces.

**Figure 6:**
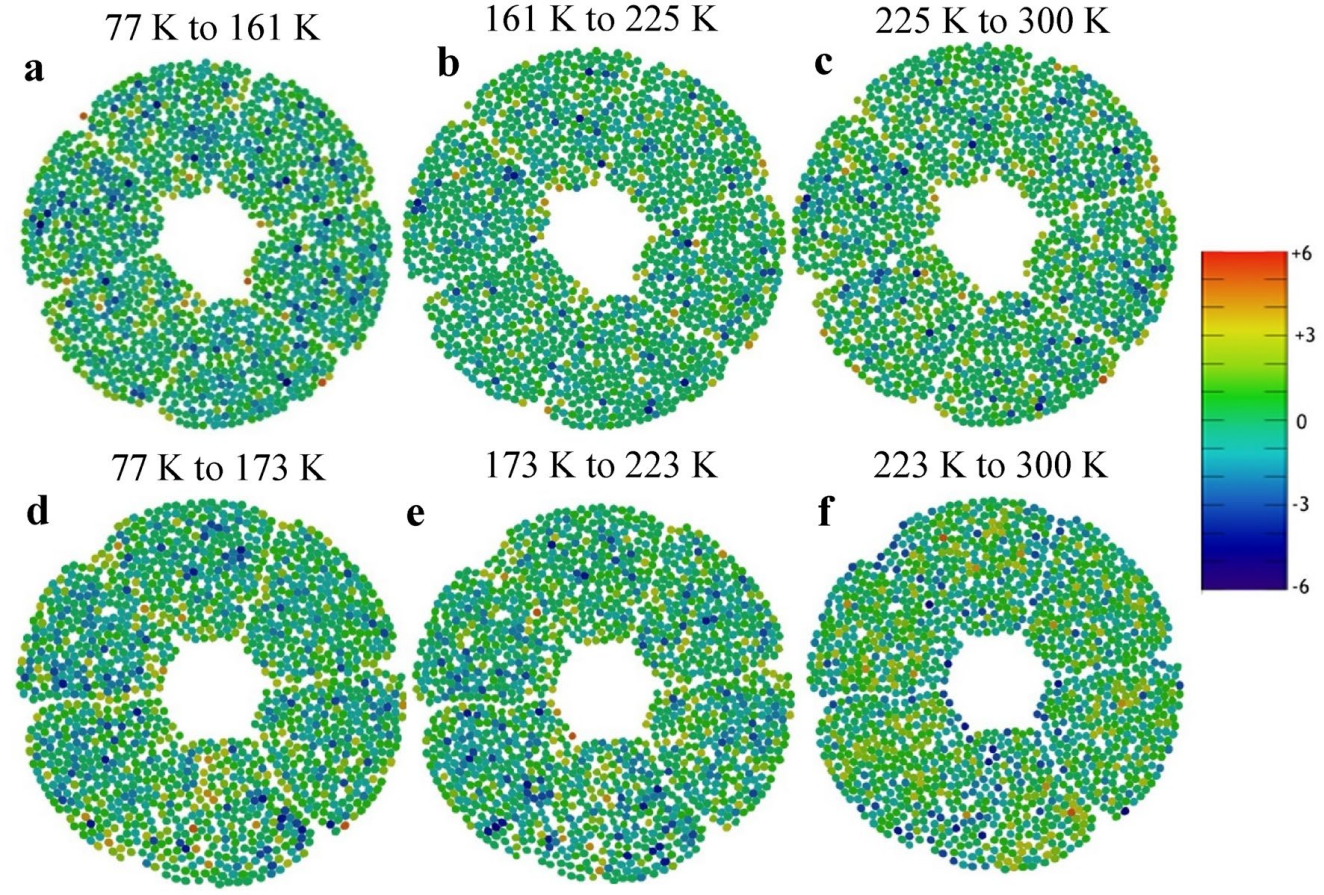
Touch number variation as a function of temperature. (**a**–**c**) Relative difference in touch number value for each superconducting bundle of the heat-treated section of conductor. A unitless colour scale is used to map and quantify the degree of change between each step in temperature. (**d**–**f**) Equivalent colour maps for the same section of a non-heat-treated conductor.

Finally, the quantification of fibre bundle ‘touch number’ provided a metric for displacement as a function of temperature, highlighting ‘hotspot’ regions where the greatest movement took place. For each fibre bundle, a value was applied based on the number of neighbouring strands it was in direct contact with. By measuring relative changes between datasets, a colour scale provided direct visualisation of strand collisions and movements through a temperature cycle. The central slice region of the reconstructed volume was evaluated for both samples. The results, shown in Fig. 6, highlighted the non-linear nature of strand movement and interaction as a function of temperature. For the non-heat-treated conductor, Fig. 6d–e show various distinct patches of reduced touch number (as bundles move apart and become isolated) as the temperature rose from liquid nitrogen levels. Significantly, however, the opposite was observed in the final change up to room temperature, with the majority of bundles in Fig. 6f experiencing an increase of 3 or more neighbouring bundle contacts. To a much lesser degree, these results were mimicked for the heat-treated sample in Fig. 6a–c. While bundle movement is still apparent with some hotspots of reduced contact initially, much fewer overall changes were observed.

## Conclusions

Through the use of a high energy, high contrast X-ray beam, the effects of a single thermal cycle were visualised for two sections of HT and NHT ITER CICC, allowing direct comparison on the effect of heat treatment to the interior superconducting fibre bundles. The advantages of synchrotron X-ray tomography were demonstrated in the study, with the ability of capturing high contrast datasets while simultaneously exposing the samples to temperature variations similar to those planned for the ITER tokamak. Post-processing allowed isolation and direct analysis of the individual fibre bundles, quantifying strand movement as a function of temperature. As expected, the effect of heat treatment significantly reduced the degree of movement with temperature.

In practice the non-heat-treated samples, whilst useful for technique development, would never be used for ITER or any future tokomak based on the ITER design. When looking at possible long term conductor stability therefore only the data from sections A1 and A2 are relevant.

Summarising these results shows that the movement of the superconducting fibre bundles is non-linear with temperature (Fig. 4a) and is affected more at the lowest temperatures we investigated, but still occurs across the whole temperature range. The strands become less circular from the centre (Fig. 5) to the outside of the conductor indicating increasing twist toward the periphery of the conductor. The number of touch points shown in Fig. 6a mirrors these findings, but also shows cables touching throughout the conductor. Touch points, non-circularity and fibre bundle movement all increase localised strain and therefore increase this risk of superconducting strand failure. Whilst the majority of the fibres imaged in this experiment remain relatively unaffected, we have however only looked at one WUCD cycle and have not taken the very high neutron flux into account.

Notwithstanding the latter point, and given the number of strands relatively unaffected by our thermal cycle, we can conclude that this CICC design is likely to survive long enough to demonstrate the principle goal of ITER to demonstrate the scientific and technological feasibility of fusion energy for peaceful use, and subsequently to bolster the global nuclear fusion industry.

However future designs of operational power stations with far more WUCD cycles might wish to take into consideration the regions of potential weakness we highlighted, in particular at the outer perimeters of the conductor and the individual sub-cable, ‘petal’ wraps (Supplementary information).

## Supplementary Information

Supplementary Information 1.

Supplementary Video 1.

Supplementary Video 2.

Supplementary Information 2.

Supplementary Information 3.

Supplementary Information 4.

Supplementary Information 5.
