# Supplementary material for: High-energy synchrotron X-ray tomography coupled with digital image correlation highlights likely failure points inside ITER toroidal field conductors
Source: Sci Rep. 2021 Nov 30;11:23141. doi: 10.1038/s41598-021-01999-5 (PMC8632903; doi:10.1038/s41598-021-01999-5)
Supplement: Supplementary file 1 — Supplementary Information 1. [file 41598_2021_1999_MOESM1_ESM.docx]

Explanation of the animated GIFs in the supplementary material.

There are four files here:

HT slice 75

HT slice 200

NHT 75 and

NHT 200

HT stands for the heat treated CICC sample. There are two fully reconstructed images for slice numbers 75 and 200.

Similarly NHT stands for the non heat treated CICC sample for slice numbers 75 and 200.

Slice numbers 75 and 200 were both towards the middle of the whole 2 cm section that has been imaged.

Both samples have been imaged at four temperatures and clearly show, albeit qualitatively, where the fibre bundles are moving. There is clearly a greater level of fibre movement in the non heat treated samples.

Two more files showing the whole depth of the sample have been uploaded.

All the videos are available for all slices if required.
