# Supplementary figures and images for: High-energy synchrotron X-ray tomography coupled with digital image correlation highlights likely failure points inside ITER toroidal field conductors
Source: Sci Rep. 2021 Nov 30;11:23141. doi: 10.1038/s41598-021-01999-5 (PMC8632903; doi:10.1038/s41598-021-01999-5)

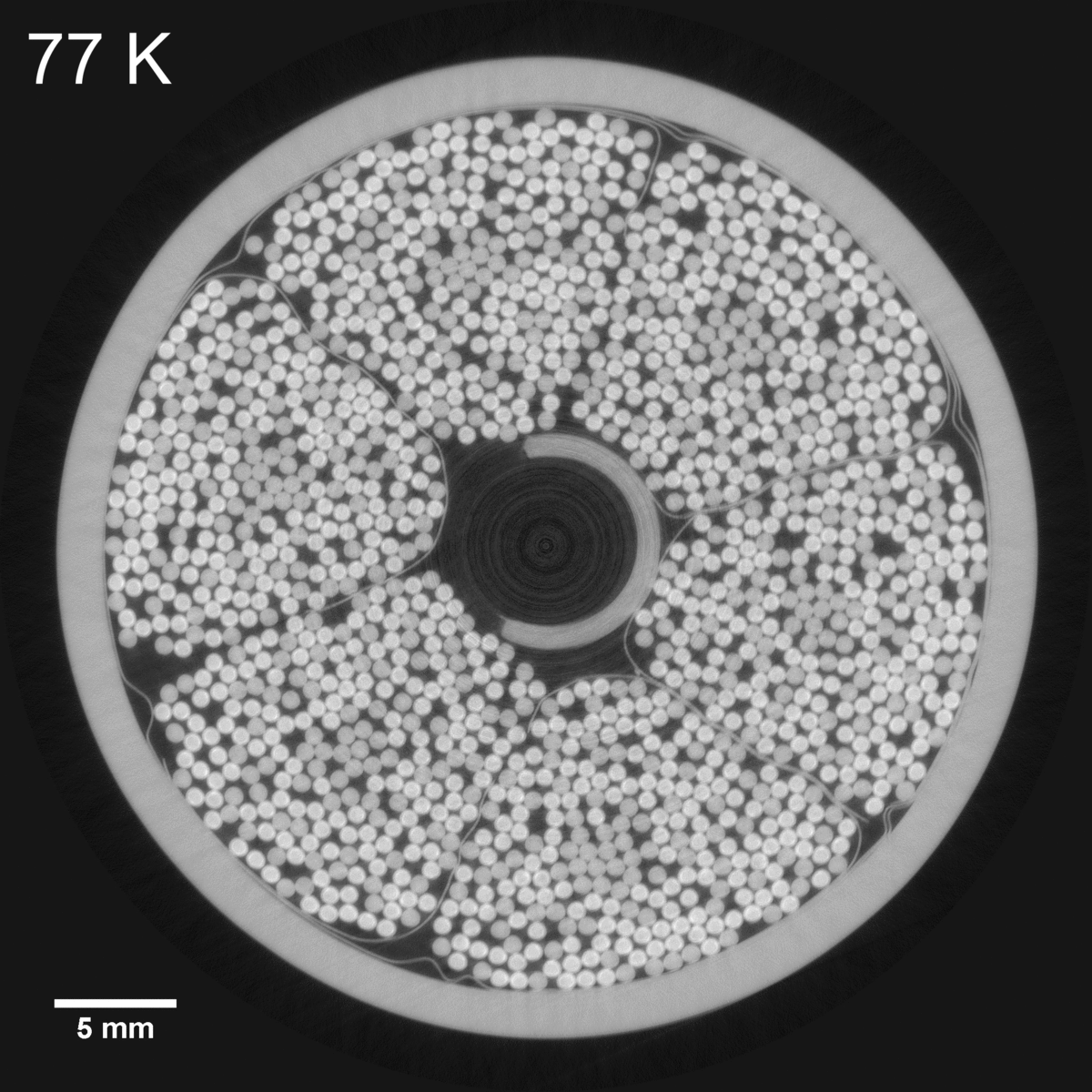

Supplement: Supplementary file 4 — Supplementary Information 2. [file 41598_2021_1999_MOESM4_ESM.gif]

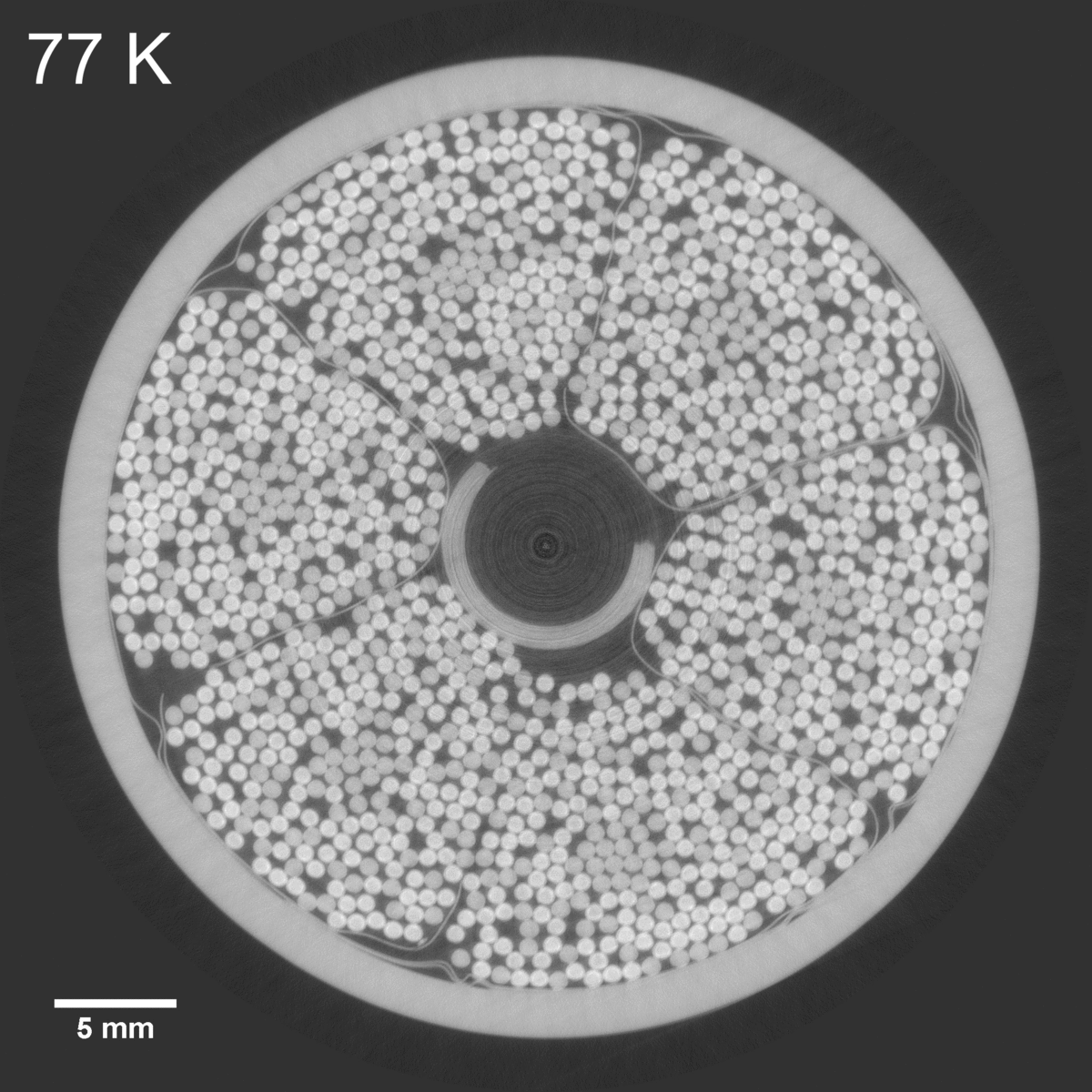

Supplement: Supplementary file 5 — Supplementary Information 3. [file 41598_2021_1999_MOESM5_ESM.gif]

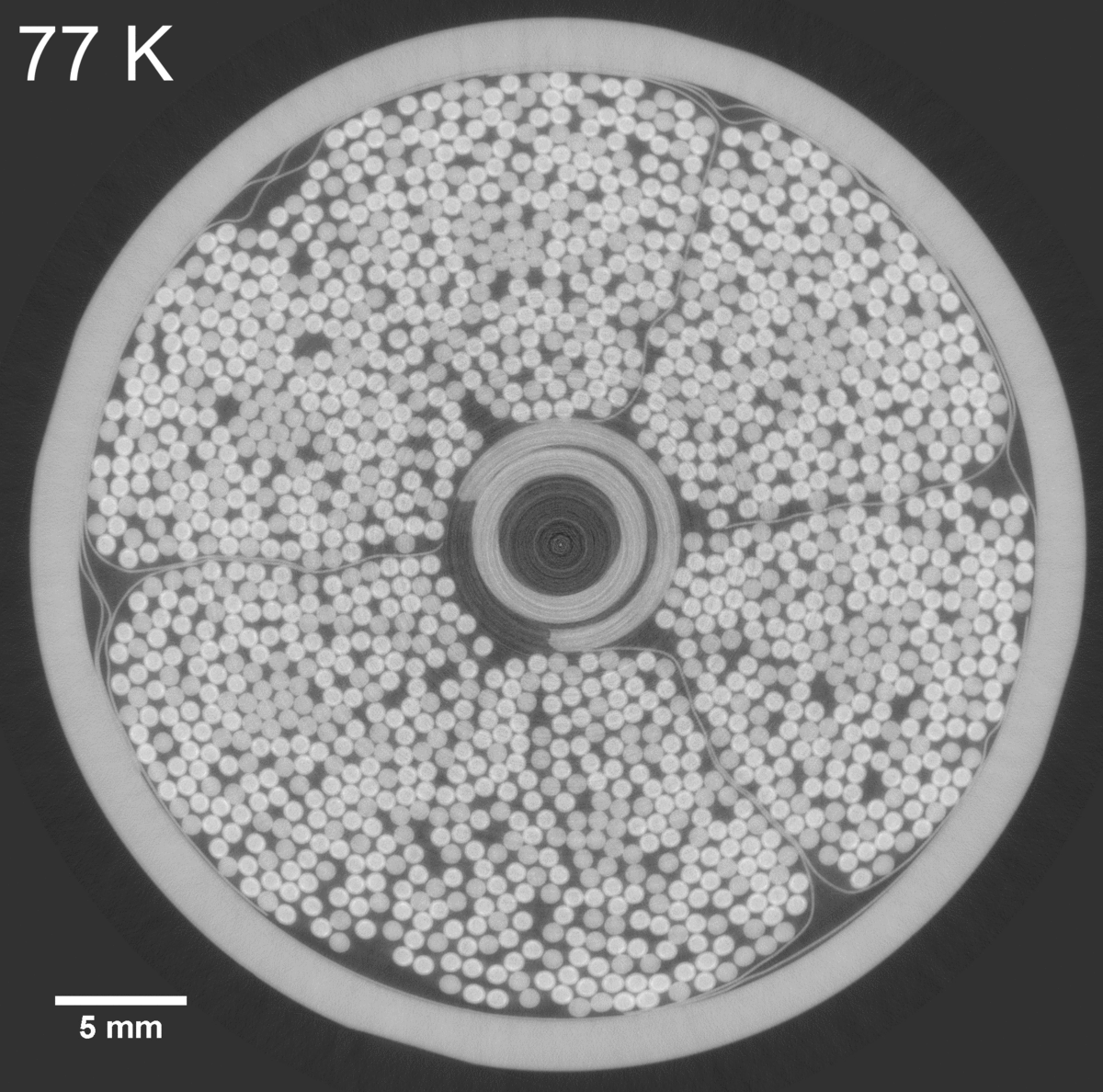

Supplement: Supplementary file 6 — Supplementary Information 4. [file 41598_2021_1999_MOESM6_ESM.gif]

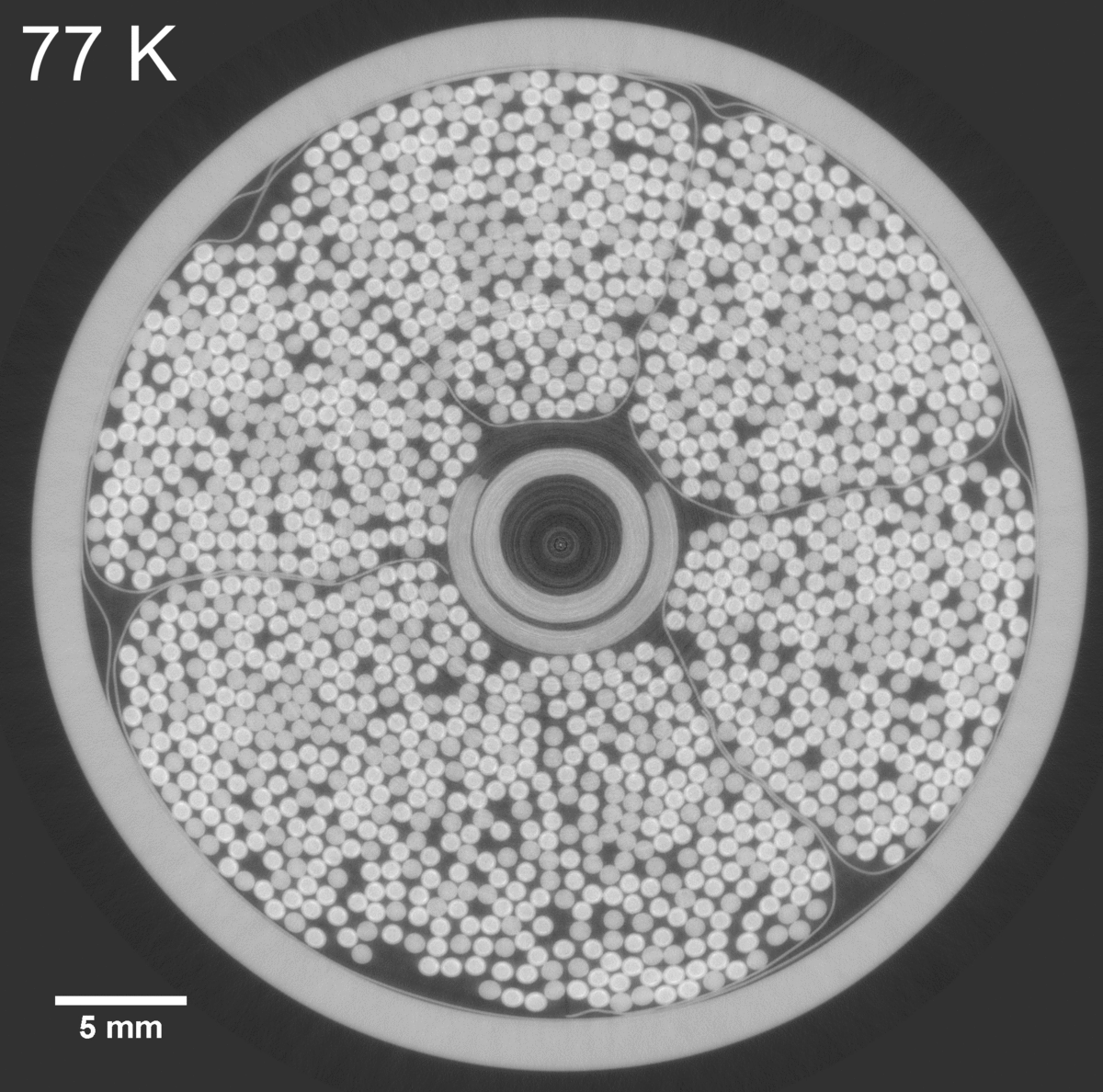

Supplement: Supplementary file 7 — Supplementary Information 5. [file 41598_2021_1999_MOESM7_ESM.gif]
